# Feral Cats and Risk for Nipah Virus Transmission

**DOI:** 10.3201/eid1207.050799

**Published:** 2006-07

**Authors:** Jonathan H. Epstein, Sohayati Abdul Rahman, Jennifer A. Zambriski, Kim Halpin, Greer Meehan, Abdul Aziz Jamaluddin, Sharifah Syed Hassan, Hume E. Field, Alex. D. Hyatt, Peter Daszak, the Henipavirus Ecology Research Group

**Affiliations:** * The Consortium for Conservation Medicine, New York, New York, USA;; † Veterinary Research Institute, Ipoh, Malaysia;; ‡ Cummings School of Veterinary Medicine at Tufts University, North Grafton, Massachusetts, USA;; § Commonwealth Scientific and Industrial Research Corporation Australian Animal Health Laboratory, Geelong, Victoria, Australia;; ¶ Ministry of Agriculture, Kuala Lumpur, Malaysia;; # Department of Primary Fisheries and Industry, Brisbane, Queensland, Australia

**Keywords:** Nipah virus, Henipavirus, Cat, Felis Catus, Pteropus, Bats, Flying Fox, Feral, Hendra virus, Public Health, letter

**To the Editor:** Nipah virus (NiV) emerged in peninsular Malaysia in 1998 and 1999 as a respiratory and neurologic disease of domestic pigs and an acute febrile encephalitic disease in humans (*1*). Nipah virus infection is associated with a case-fatality ratio of 40% to 76% in humans (*1**,**2*). Cats (*Felis catus*) were infected with NiV at the site of the outbreak in northern Malaysia (*3*). Experimental studies have shown that cats are susceptible to Hendra virus and NiV (*4**,**5*). Infected cats shed NiV through the nasopharynx and in urine while viremic, and 1 (of 2) recovered from experimental NiV infection with a high neutralizing antibody titer (>256) within 21 days (*5*).

Fruit bats of the genus *Pteropus* are believed to be the reservoir for NiV in Malaysia (*6*). In June 2000, NiV was isolated from partially eaten fruit and from the urine of *Pteropus hypomelanus* in the village of Air Batang on Tioman Island, Peninsular Malaysia (*7*). Although humans live in close proximity to these bats, no evidence for local human exposure to NiV has been seen (*8*). In contrast, epidemiologic evidence from recent NiV outbreaks in Bangladesh suggests that direct infection from pteropid bats may occur, possibly when bats are pregnant (*2**,**9*).

Despite limited contact with bats, residents and visitors to Air Batang have ample opportunity for close contact with feral cats, which are often fed and sometimes housed by residents. Cats have been observed under trees that are occupied by roosting fruit bats in Air Batang. NiV could be transmitted from bats to cats through urine and then among cats oronasally, given their gregarious nature, which frequently includes mutual grooming. Cats are also frequently seen in close contact with humans in restaurants, on the tables, and in food preparation areas, where they are fed. If NiV is also present in bat fetal tissues, cats could become infected through contact with or by eating these tissues after mass births among bats.

We tested feral cats from Air Batang for neutralizing antibodies to NiV to determine whether cats might play a role in the zoonotic transmission of Nipah virus. Fifty bats were captured from Air Batang and tested for NiV and neutralizing antibodies to NiV as part of a long-term NiV surveillance study (A. Rahman, unpub. data).

Thirty-two cats were caught July 12–19, 2004, in a 200-m radius of a bat colony. Cats were anesthetized, and 3.0 mL blood was collected from the jugular vein or medial saphenous vein. Serum was allowed to separate at 4°C for 24 hours and was then further separated and frozen in liquid nitrogen. Serum was tested by serum neutralization test (SNT), which is considered the reference standard for serologic assays, at the Australian Animal Health Laboratory, Geelong, Australia, as described (*5**,**10*).

The time of year was similar to the time when NiV was isolated from bats in 2000; however, none of the 32 cats (18 males, 14 females; 25 adults, 7 juveniles [<1 year of age]) had detectable antibodies to NiV on SNT. All cats appeared healthy except for 1 adult that was markedly jaundiced. The period of the study did not overlap the seasonal gestation period of *P. hypomelanus*, and none of the adult female bats tested (n = 20) were pregnant. Although attempts to isolate virus from bat urine and saliva were unsuccessful (A. Rahman, unpub. data), 7 (14%) of 50 bats, including 1 (8%) of 13 post-weaning juveniles (≈4 months to 2 years of age) had neutralizing antibodies (all >32) to NiV on SNT, which suggests that virus had circulated in the colony since 2000.

Our finding of no seropositive cats may be explained in 3 ways: 1) feral cats are rarely, if at all, exposed to NiV in nature; 2) the death rate from NiV infection in cats is so high that few or none survive with immunity; or 3) our sample size was too small to detect a seropositive cat. We believe that the first hypothesis is most likely. A low incidence of NiV infection in this population of bats (95% confidence interval for 0 of 50 bats, 0.00–0.71), combined with a short viremic period, would make transmission between bats and cats unlikely. However, if transmission occurred, we would expect to find some cats with a detectable titer (*5*). While the exact age of the cats in this survey was unknown, 25 (78%) of 32 were adults (>1 year of age) and may have been in Air Batang either in 2000, when NiV was isolated from bats, or during a more recent outbreak. We conclude that exposure of feral or peridomestic cats to Nipah virus on Tioman Island is rare and that the risk for zoonotic transmission is low.

## References

[1] Chua KB, Bellini W, Rota P, Harcourt B, Tamin A, Lam S, Nipah virus: A recently emergent deadly paramyxovirus. Science. 2000;288:1432–5. 10.1126/science.288.5470.1432 10827955

[2] Hsu VP, Hossain MJ, Parashar UD, Mohammed MA, Ksiazek TG, Kuzmin I, Nipah virus encephalitis reemergence, Bangladesh. Emerg Infect Dis. 2004;10:2082–7. 15663842 10.3201/eid1012.040701 PMC3323384

[3] Mohd Nor MN, Gan CH, Ong BL. Nipah virus infection of pigs in peninsular Malaysia. Rev Sci Tech. 2000;19:160–5. 11189713 10.20506/rst.19.1.1202

[4] Westbury HA, Hooper PT, Brouwer SL, Selleck PW. Susceptibility of cats to equine morbillivirus. Aust Vet J. 1996;74:132–4. 10.1111/j.1751-0813.1996.tb14813.x 8894019

[5] Middleton DJ, Westbury HA, Morrissy CJ, van der Heide BM, Russell GM, Braun MA, Experimental Nipah virus infection in pigs and cats. J Comp Pathol. 2002;126:124–36. 10.1053/jcpa.2001.0532 11945001

[6] Yob JM, Field H, Rashdi AM, Morrissy C, van der Heide B, Rota P, Nipah virus infection in bats (order *Chiroptera*) in peninsular Malaysia. Emerg Infect Dis. 2001;7:439–41. 11384522 10.3201/eid0703.010312 PMC2631791

[7] Chua KB, Koh C, Hooi P, Wee K, Khong J, Chua B, Isolation of Nipah virus from Malaysian island flying foxes. Microbes Infect. 2002;4:145–51. 10.1016/S1286-4579(01)01522-2 11880045

[8] Chong HT, Chong TT, Goh KJ, Lam SK, Chua KB. The risk of human Nipah virus infection directly from bats (*Pteropus hypomelanus*) is low. Neurology Asia [serial on the Internet]. 2003 [cited 2006 May 16]. Available from http://www.neurology-asia.org/articles/20031_031.pdf

[9] ICDDR. B. Nipah virus outbreak from date palm juice. Health and Science Bulletin [serial on the Internet]. 2005 Dec [cited 2006 May 16]. Available from http://www.icddrb.org/pub/publication.jsp?classificationID=56&pubID=6590

[10] Daniels P, Ksiazek T, Eaton BT. Laboratory diagnosis of Nipah and Hendra virus infections. Microbes Infect. 2001;3:289–95. 10.1016/S1286-4579(01)01382-X 11334746

